# Treatment of granuloma annulare with tofacitinib

**DOI:** 10.1111/ajd.13875

**Published:** 2022-05-23

**Authors:** Xavier Bosch‐Amate, Laura Serra‐García, Francesc Alamon‐Reig, Ignasi Marti‐Marti, Javier Gil‐Lianes, Priscila Giavedoni, José M. Mascaró

**Affiliations:** ^1^ Department of Dermatology Hospital Clinic of Barcelona, University of Barcelona Barcelona Spain

Dear Editor,

Granuloma annulare (GA) is a granulomatous, idiopathic, inflammatory skin disorder characterized by the formation of papules and plaques with annular and acral distribution.^1^ GA is often limited and self‐resolving, but in some cases, it can be generalized and refractory to treatments.^1^ New advances in the pathophysiology of GA have favoured Janus kinase (JAK) inhibitors as a promising therapeutic option.^1^, ^2^ Here, we report three cases of resistant generalized GA successfully treated with tofacitinib.

The first was a 69‐year‐old woman with a 6‐year history of generalized GA who had been treated with oral corticosteroids, retinoic and tranexamic acid, indomethacin, hydroxychloroquine and methotrexate without improvement or intolerance, and the second a 73‐year‐old woman with a history of dyslipidemia receiving simvastatin treatment who was diagnosed with generalized GA in 2016. She had received oral corticosteroids, hydroxychloroquine, pentoxifylline and dapsone, without improvement (Figure 1a). The third patient was a 64‐year‐old man with a 3‐year evolution of a generalized GA who had received oral corticosteroids, phototherapy, methotrexate, hydroxychloroquine and adalimumab with partial response. Furthermore, he had begun experiencing flares of bilateral uveitis in the context of skin outbreaks (Figure 1c).

**FIGURE 1:**
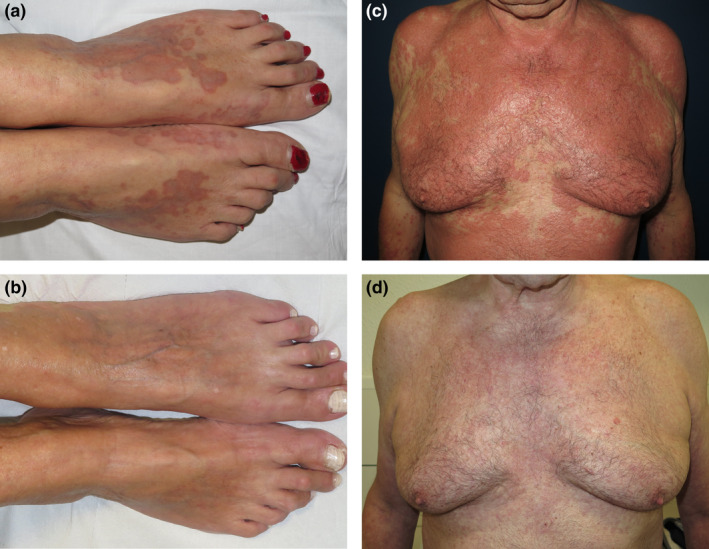
Generalized granuloma annulare. Baseline and after tofacitinib treatment. (a) and (b) (patient 12). Granuloma annulare lesions on the dorsal aspect of both feet before and 8 months after initiating treatment. (c) and (d) (patient 13). Granuloma annulare lesions on anterior chest before and 6 months after initiating treatment

In all three cases, the diagnosis was confirmed histologically and due to difficulties in management, treatment with off‐label oral tofacitinib was requested and approved by the therapeutic committee. After the three patients gave written informed consent, tofacitinib was initiated at a dose of 5 mg twice daily. The three patients showed complete response after 4, 8 and 6 months, respectively (Figure 1b and d), without relapses after dose tapering or suspension of the treatment. The characteristics of all the reported GA patients treated with tofacitinib are summarized in Table 1.

**TABLE 1:** Patients with granuloma annulare treated with tofacitinib

Patient	Author/year	Gender/age	Clinic GA type	Basal involved BSA (%)	Disease duration	Previous systemic therapies	Tofacitinib treatment	Adverse effects	Outcome and follow‐up
1	Damsky/2020^2^	F/66	Generalized	NR	6 years	Hydroxychloroquine, SC, UV, doxycycline, cyclosporine, isotretinoin	5 mg twice daily	None	CR at month 6. Response sustained with tofacitinib until the writing of the case
2	Damsky/2020^5^	M/69	Localized	NR	1 year	None	Topical 2% ointment twice daily	None	Near resolution of treated lesions at week 15. No improvement in untreated lesions. No follow‐up described
3	Durgin/2020^6^	F/46	Generalized	10	2 years	Hydroxychloroquine, dapsone	Topical 2% ointment twice daily	None	PR (BSA improvement of 90% from baseline) at week 12 applying only the treatment to the lesions of the right arm. Without relapses in 2 month of follow‐up
4	Wang/2021^1^	F/60	Generalized	7	6 years	SC, pentoxifylline	5 mg twice daily	None	PR (BSA improvement of 71.4% from baseline) at month 6. No follow‐up described
5	Wang/2021^1^	F/68	Generalized	30	6 years	Hydroxychloroquine, SC, cyclosporine, antibiotics, UV	5 mg twice daily	None	CR at month 6. No follow‐up described
6	Wang/2021^1^	F/64	Generalized	10	6 years	Pentoxifylline	5 mg twice daily	None	PR (BSA improvement of 60% from baseline) at month 6. No follow‐up described
7	Wang/2021^1^	M/53	Generalized	18	10 years	Hydroxychloroquine, antibiotics, UV	5 mg twice daily	None	CR at month 6. No follow‐up described
8	Wang/2021^1^	F/65	Generalized	8	15 years	None	5 mg twice daily	None	CR at month 6. No follow‐up described
9	McPhie/2021^4^	F/78	Generalized	NR	6 years	UV, methotrexate, ustekinumab	5 mg twice daily	None	Almost CR at month 9. No follow‐up described
10	McPhie/2021^4^	F/59	Generalized	NR	10 years	UV, ustekinumab	5 mg twice daily	None	PR (degree of improved NR) at week 4. No follow‐up described
11	New case reported in this manuscript	F/69	Generalized	12	5 years	Hydroxychloroquine, SC, retinoic acid, tranexamic acid, indomethacin, methotrexate	5 mg twice daily	None	CR at month 4. Response sustained 4 months after suspension and 11 months after starting dose tapering
12	New case reported in this manuscript	F/73	Generalized	10	5 years	Hydroxychloroquine, SC, pentoxifylline, dapsone	5 mg twice daily	None	CR at month 8. Response sustained 6 months after suspension and 17 months after starting dose tapering
13	New case reported in this manuscript	M/64	Generalized	45	3 years	Hydroxychloroquine, SC, UV, methotrexate, adalimumab	5 mg twice daily	None	CR at month 6. Response sustained 10 month after suspension

Abbreviations: BSA—body surface area; CR—complete response; F—female; M—male; GA—granuloma annulare; NR—not reported; PR—partial response; SC—systemic corticosteroids; UV—narrowband ultraviolet B phototherapy.

JAK inhibitors represent a promising approach for cutaneous granulomatous disorders.^2^ Damsky et al demonstrated that JAK–STAT signalling was constitutively activated in GA lesional macrophages, and that tofacitinib treatment induced histologic clearance of granulomas and downregulation of the JAK–STAT pathway.^2^ Further, Wang et al recently found that IFN‐γ, oncostatin M, IL‐21 and IL‐15, four important cytokines that signal via the JAK–STAT pathway, are upregulated in GA skin lesions.^1^ Further, Min et al identified a significant upregulation of inflammatory T‐helper cell types 1 and 2, and Janus kinase immune pathways in GA patients.^3^

Tofacitinib is a potent inhibitor of JAK 1 and 3 in human cells. Currently, it is approved for the treatment of rheumatoid arthritis, psoriatic arthritis, juvenile idiopathic arthritis and ulcerative colitis with a recommended dose of 5 mg twice daily. Recent data from post‐marketing surveillance have shown an increased risk of death, major adverse cardiovascular events, malignancies and thrombosis related to tofacitinib prompting the FDA to issue a safety communication. We found eight previous reports in the literature of patients treated with oral tofacitinib for GA^1^, ^2^, ^4^ and two reports of treatment with topical tofacitinib.^5^, ^6^ All patients improved ranging from a partial response of 60% to complete remission. Topical treatment showed improvement between weeks 12 and 15, while oral treatment took about 6 months. None of the cases described have reported any adverse effects. In addition, other JAK inhibitors such as baricitinib and upadacitinib have recently been used in two generalized GA patients with good results.^7^, ^8^

In conclusion, advances in GA pathophysiology have allowed the introduction of JAK inhibitors as a new treatment option. To our knowledge, there are only about a dozen reports of GA patients treated with tofacitinib. So far, it has shown an excellent response rate, with both oral and topical administration. Further studies are necessary to assess the safety and the long‐term remission of GA patients treated with tofacitinib, and to study the efficacy of other JAK inhibitors in these patients.

## FUNDING INFORMATION

None.

## CONFLICT OF INTEREST

The authors declare that there is no conflict of interest.
